# The Molecular and Microenvironmental Landscape of Glioblastomas: Implications for the Novel Treatment Choices

**DOI:** 10.3389/fnins.2020.603647

**Published:** 2020-11-25

**Authors:** Federica Di Cintio, Michele Dal Bo, Lorena Baboci, Elena De Mattia, Maurizio Polano, Giuseppe Toffoli

**Affiliations:** ^1^Experimental and Clinical Pharmacology Unit, Centro di Riferimento Oncologico di Aviano, Istituto di Ricovero e Cura a Carattere Scientifico (IRCCS), Aviano, Italy; ^2^Department of Life Sciences, University of Trieste, Trieste, Italy

**Keywords:** GBM, tumor microenvironment, immune checkpoint inhibitors, tyrosine kinase inhibitors, CAR-T, treatment resistance

## Abstract

Glioblastoma (GBM) is the most frequent and aggressive primary central nervous system tumor. Surgery followed by radiotherapy and chemotherapy with alkylating agents constitutes standard first-line treatment of GBM. Complete resection of the GBM tumors is generally not possible given its high invasive features. Although this combination therapy can prolong survival, the prognosis is still poor due to several factors including chemoresistance. In recent years, a comprehensive characterization of the GBM-associated molecular signature has been performed. This has allowed the possibility to introduce a more personalized therapeutic approach for GBM, in which novel targeted therapies, including those employing tyrosine kinase inhibitors (TKIs), could be employed. The GBM tumor microenvironment (TME) exerts a key role in GBM tumor progression, in particular by providing an immunosuppressive state with low numbers of tumor-infiltrating lymphocytes (TILs) and other immune effector cell types that contributes to tumor proliferation and growth. The use of immune checkpoint inhibitors (ICIs) has been successfully introduced in numerous advanced cancers as well as promising results have been shown for the use of these antibodies in untreated brain metastases from melanoma and from non-small cell lung carcinoma (NSCLC). Consequently, the use of PD-1/PD-L1 inhibitors has also been proposed in several clinical trials for the treatment of GBM. In the present review, we will outline the main GBM molecular and TME aspects providing also the grounds for novel targeted therapies and immunotherapies using ICIs for GBM.

## Introduction

Glioblastoma (GBM) is the most common and aggressive primary CNS tumor (Stupp et al., 2009; Louis et al., 2016; Mendes et al., 2018; Altmann et al., 2019; Ostrom et al., 2019) and it has been included in the group of diffuse astrocytic and oligodendroglial tumors by the 2016 CNS WHO (Louis et al., 2016). It is thought that genetic alterations affecting neuroglial stem or progenitors cells give origin to GBM. The incidence of this tumor seems to increase with age; in fact, 62 years is the median age at diagnosis. Males are affected by GBM tumors 1.7 fold more often than females. According to the presence of mutations in the isocitrate dehydrogenase (*IDH*) 1 and *IDH2* genes GBM is subdivided by the WHO into two major types. More than 90% of GBM cases is constituted by GBM with wild type *IDH* (Louis et al., 2016). Clinically, grade IV lesions (namely primary GBM) are presented *de novo* by the majority of patients, while progression from a less aggressive form of WHO grade II diffuse astrocytomas and WHO grade III anaplastic astrocytomas (i.e., secondary GBM) is shown by a small fraction of patients (5–10%; Ohgaki and Kleihues, 2013; Louis et al., 2016). Primary GBM and secondary GBM differ in prognosis and age of onset. As for overall survival (OS) (Doetsch et al., 1999; Louis et al., 2016), primary GBM is typically diagnosed at older age and has a worse prognosis while secondary GBM are less common and affect people under the age of 45; also they develop into low-grade astrocytoma and are associated with better prognosis (Doetsch et al., 1999; Brennan et al., 2013; Ohgaki and Kleihues, 2013; Louis et al., 2016).

Standard of care first-line treatment is constituted by maximal surgical resection (complete resection is performed quite rarely because of the presence of diffuse infiltrations), followed by radiotherapy with concomitant and adjuvant chemotherapy such as the oral alkylating agent, temozolomide (TMZ). Upon this treatment combination GBM show a median OS of about 15 months (Canoll and Goldman, 2008; Stupp et al., 2009; Ohgaki and Kleihues, 2013; Levine et al., 2015).

The increase of patient survival is small and tumors invariably recur after TMZ (Canoll and Goldman, 2008; Stupp et al., 2009; Ohgaki and Kleihues, 2013; Levine et al., 2015). Following the first recurrence, treatment choices can be represented by further surgical resection when possible, or conventional chemotherapy, e.g., TMZ (with different dosing schedules) or nitrosoureas, or treatment with the anti-vascular endothelial growth factor (VEGF) agent, bevacizumab, or the use of the low-intensity alternating electric fields (TTFields). However, these treatments have not achieved significant improvements in survival (Canoll and Goldman, 2008; Stupp et al., 2009; Chamberlain and Johnston, 2010; Stupp et al., 2012; Ohgaki and Kleihues, 2013; Stupp and Hegi, 2013; Chamberlain, 2015). Moreover, the tyrosine kinase inhibitor (TKI) regorafenib has been introduced in the treatment of recurrent GBM (Lombardi et al., 2019).

A detailed characterization of the GBM-associated molecular signatures has made possible the development of novel therapies, including the use of TKIs (Friedman et al., 2009; Quant et al., 2009; Brennan et al., 2013; Wang et al., 2016; Lombardi et al., 2019). On the other hand, based on the results obtained in the context of other tumors (Brahmer et al., 2010; Eder and Kalman, 2014; Larkin et al., 2015; Weber et al., 2015; Kessler et al., 2018; Stathias et al., 2018), the use of programmed cell death protein (PD-1) receptor/programmed death ligand 1 (PD-L1) inhibitors has been suggested for gliomas, including GBM (Motzer et al., 2015; Goldberg et al., 2016; Reck et al., 2016; Schwartz et al., 2016; Reiss et al., 2017; Reardon et al., 2018; Cloughesy et al., 2019; Schalper et al., 2019).

In the present review, we will outline the principal GBM molecular and tumor microenvironment (TME) aspects providing also the grounds for novel targeted therapies and immunotherapy approaches using ICIs for the treatment of GBM affected patients.

## Genomic Landscape of GBM

Specific molecular signatures of GBM have been identified through the introduction of next generation sequencing methods, in particular in untreated GBM tumors. It has been found mutations of several genes in GBM including phosphatase and tensin homolog (*PTEN*), tumor suppressor P53 (*TP53*), epidermal growth factor receptor (*EGFR*), phosphatidylinositol-4,5-bisphosphate 3-kinase catalytic subunit alpha (*PIK3CA*), phosphatidylinositol 3-kinase regulatory subunit alpha (*PIK3R1*), platelet derived growth factor receptor alpha (*PDGFRA*), retinoblastoma 1 (*RB1*), neurofibromin 1 (*NF1*), gamma-aminobutyric acid receptor subunit alpha-6 (*GABRA6*), *IDH1*, mutS homolog 2 (*MSH2*), mutS homolog 6 (*MSH6*), mutL homolog 1 (*MLH1*), and PMS1 homolog 2 (*PMS2*). Furthermore, several hotspot mutations have been found, like the *IDH1* R132H mutation, the B-Raf proto-oncogene (*BRAF*) V600E mutation (Zhao et al., 2009; Kloosterhof et al., 2011; Kannan et al., 2012; Schwartzentruber et al., 2012; Brennan et al., 2013; Eder and Kalman, 2014; Ceccarelli et al., 2016; Wang et al., 2016; Kessler et al., 2018; Stathias et al., 2018; D’Angelo et al., 2019).

Glioblastoma cases characterized by the presence of mutations in DNA mismatch repair (MMR) genes, e.g., *MSH2*, *MSH6*, *MLH1*, and *PMS2* have been suggested to be defined as having a hypermutated profile (Hunter et al., 2006; Cahill et al., 2007; Greenman et al., 2007; Tcga, 2008; Yip et al., 2009; Brennan et al., 2013; Daniel et al., 2019).

Frequent amplification events found in GBM concern chromosome 7 [*EGFR*/ MET proto-oncogene (*MET*)/ cyclin dependent kinase 6 (*CDK6*)], chromosome 12 [cyclin dependent kinase 4 (*CDK4*)/, mouse double minute 2 homolog (*MDM2*)], and chromosome 4 (*PDGFRA*). Gains of the genes SRY-box transcription factor 2 (*SOX2*), MYCN proto-oncogene (*MYCN*), cyclin D1 (*CCND1*), and cyclin E2 (*CCNE2*) have also been found (Hunter et al., 2006; Cahill et al., 2007; Kuttler and Mai, 2007; Parsons et al., 2008; Tcga, 2008; Yip et al., 2009; Brennan et al., 2013; Sanborn et al., 2013; Zheng et al., 2013; Furgason et al., 2015). Frequent deletions in GBM include deletions in cyclin-dependent kinase inhibitor 2A/B (*CDKN2A/B*), deletions of 6q26 in which the minimal deleted region seems to include the QKI, KH domain containing RNA binding (*QKI*) gene, and single gene deletions of low-density lipoprotein receptor-related protein 1B (*LRP1B*), neuronal PAS domain protein 3 (*NPAS3*), limbic system associated membrane protein (*LSAMP*), SET and MYND domain-containing protein 3 (*SMYD3*) genes (Kamiryo et al., 2002; Hunter et al., 2006; Cahill et al., 2007; Tcga, 2008; Yip et al., 2009; Moreira et al., 2011; Chen et al., 2012; Mizoguchi et al., 2012; Brennan et al., 2013; Nobusawa et al., 2014; Tabouret et al., 2015; Yang et al., 2016).

Repeatedly, *EGFR* mutations have been found associated with regional gene amplification (Ekstrand et al., 1991; Jaros et al., 1992; Schlegel et al., 1994; Dunn et al., 2012; Brennan et al., 2013; Cominelli et al., 2015). Remarkably, the aberrant exon 1–8 junction of epidermal growth factor receptor variant III (*EGFRvIII*) was found expressed in a relevant proportion of cases. Additional recurrent non-canonical *EGFR* transcript forms were also observed (Ekstrand et al., 1991; Jaros et al., 1992; Nishikawa et al., 1994; Tcga, 2008; Brennan et al., 2013; Cominelli et al., 2015). The O-6-methylguanine-DNA methyltransferase (*MGMT*) locus has been found methylated in about 50% of GBM cases (Esteller et al., 2000; Paz et al., 2004; Hegi et al., 2005; Tcga, 2008; Zawlik et al., 2009; Malmstrom et al., 2012; Reifenberger et al., 2012; Armstrong et al., 2013; Brennan et al., 2013; Wiestler et al., 2013; Wick et al., 2014, 2018; Cominelli et al., 2015).

Recent studies have defined the evolution of tumor cells in GBM cases undergoing therapy as a process of clonal replacement where a fraction of tumor cells is eliminated by the treatment while clones of resistant cells are positively selected (Tcga, 2008; Brennan et al., 2013; Wang et al., 2016). Specifically, intratumoral heterogeneity, with the presence of resistant subclones, both in low grade and high grade glioma is frequently associated with treatment failure. Although a clearly defined pattern of tumor evolution has not yet been described in GBM, *TP53* gene mutations have been recently proposed as a marker of subclonal heterogeneity (Tcga, 2008; Brennan et al., 2013; Wang et al., 2016).

Glioblastoma evolution is highly branched, specific alterations and evolutionary patterns frequently occurring depending on the treatment. There is no linear link between the dominant clone at diagnosis and the dominant clone at relapse (Tcga, 2008; Brennan et al., 2013; Wang et al., 2016). Remarkably, genes such as *TP53, EGFR, PDGFRA* are frequently subjected to a process of mutational switching where a mutated version of a gene, found at diagnosis, is replaced by another mutated version of the same gene at relapse (Tcga, 2008; Brennan et al., 2013; Wang et al., 2016). Hypermutated tumors, which are highly enriched for mutations at CpG dinucleotides, generally harbor mutations in *MMR* pathway genes, most commonly in *MSH6*. These *MMR* alterations have been thought to be associated with putative mutagenic mechanisms of TMZ treatment (Hunter et al., 2006; Cahill et al., 2007; Tcga, 2008; Yip et al., 2009; Brennan et al., 2013; Wang et al., 2016).

A gene expression-based molecular classification has been proposed to integrate somatic mutation and DNA copy number data (Verhaak et al., 2010; Behnan et al., 2019). According to this classification, GBM cases were subdivided in proneural, neural, classical and mesenchymal subtypes. These different subtypes have been associated with gene signatures of normal brain cell types of different neural lineages. Moreover, GBM cases included in the different subtypes have also been associated with a different pathogenesis with GBM clones developing as the result of different causes and/or from different cell type of origin. However, further studies, also investigating glioma stem cells, have been able to identify three subtypes: proneural, mesenchymal and classical subtypes (Verhaak et al., 2010; Behnan et al., 2019).

According to the first proposed classification, GBM cases belonging to the classical subtype show in about the 100% of cases the chromosome 7 amplification paired with chromosome 10 loss. This event is also very frequent in the totality of GBM cases. High-level of *EGFR* amplification has been observed in 97% of cases belonging to the classical subtype, whereas this alteration has been infrequently found in the other GBM subtypes. Moreover, in association with frequent EGFR alteration, a lack of *TP53* mutations has been found in a subset of the classical subtype (Tcga, 2008; Verhaak et al., 2010; Brennan et al., 2013; Wang et al., 2016). The focal 9p21.3 homozygous deletion, targeting the *CDKN2A* gene, has been also frequently found in the classical subtype, in the 94% of cases belonging to this subtype found associated with *EGFR* amplification. The homozygous 9p21.3 deletion has been also found mutually exclusive with aberrations in genes belonging to the RB1 pathway such as *RB1*, *CDK4* and cyclin-D2 (*CCDN2)*, thus suggesting that in the cases with focal *EGFR* amplification the *CDKN2A* deletion is the sole alteration affecting the RB1 pathway. GBM cases belonging to the classical subtype are also characterized by the high expression of genes belonging to the notch homolog 1, translocation-associated (NOTCH) pathway such as neurogenic locus notch homolog-3 (*NOTCH3*), jagged-1 (*JAG1*) and lunatic fringe (*LFNG*), sonic hedgehog pathway such as smoothened (*SMO*), growth arrest-specific protein 1 (*GAS1*) and zinc finger protein GLI1 (*GLI1*) and the neural precursor and stem cell marker nestin (NES) pathway (Tcga, 2008; Verhaak et al., 2010; Brennan et al., 2013; Wang et al., 2016).

Glioblastoma cases belonging to the proneural subtype were found to be mainly characterized by alterations of *PDGFRA* and point mutations of *IDH1*. The focal amplification at the locus 4q12 harboring *PDGFRA* was associated with high levels of *PDGFRA* gene expression and the frequent presence of mutations in the *PDGFRA* gene. The great majority of *IDH1* mutations has been found in GBM cases belonging to the proneural subtype. Of note, they have been found to be generally mutually exclusive to *PDGFRA* alterations. Loss of heterozygosity and mutations of the *TP53* gene have been found to be frequent events in the proneural subtype. *PIK3CA/PIK3R1* mutations have also been found in the proneural subtypes in cases without *PDGFRA* abnormalities. The proneural group has been found to be characterized also by the high expression of genes other than *PDGFRA* that characterize the oligodendrocytic development such as oligodendrocyte transcription factor (*OLIG2*) and homeobox protein nkx-2.2 (*NKX2-2*). This group has been found also characterized by the expression of proneural development genes such as *SOX* family genes and achaete-scute homolog 1 (*ASCL1*), doublecortin (*DCX*), delta-like 3 (*DLL3*), transcription factor 4 (*TCF4*; Tcga, 2008; Verhaak et al., 2010; Brennan et al., 2013; Wang et al., 2016).

Glioblastoma cases belonging to the neural subtype were characterized by the expression of genes well-known as neuron markers such as *GABRA1*, neurofilament light chain (*NEFL*), synaptotagmin-1 (*SYT1*) and solute carrier family 12 member 5 (*SLC12A5*). GBM cases belonging to the neural subtype show an enrichment in genes involved in neuron protection and in axon and synaptic transmission (Tcga, 2008; Verhaak et al., 2010; Brennan et al., 2013; Wang et al., 2016).

Glioblastoma cases belonging to the mesenchymal subtype are frequently characterized by the presence of focal hemizygous deletions at 17q11.2 region encompassing the *NF1* gene. This has been frequently associated with low *NF1* expression levels. Moreover, mutations at the *NF1* gene have been found in GBM cases belonging to the mesenchymal subgroup. Concomitant *PTEN* mutations have also been found in mesenchymal subgroup cases carrying *NF1* mutations (Tcga, 2008; Verhaak et al., 2010; Brennan et al., 2013; Wang et al., 2016). GBM cases belonging to the mesenchymal subtype are also characterized by the expression of mesenchymal markers such as chitinase-3-like protein 1 (*CHI3L1*) and *MET*. It has been thought that the higher activity of mesenchymal and astrocytic markers such as *CD44* and *MERKT* is linked to an epithelial-to-mesenchymal transition proper of dedifferentiated and transdifferentiated tumors. Finally, GBM cases belonging to the mesenchymal subtype are also characterized by the high expression of genes belonging to the TNF superfamily pathway and NF-kB pathway such as tumor necrosis factor receptor type 1-associated death domain (*TRADD*), *RELB* and TNF receptor superfamily member 1A (*TNFRSF1A*) (Tcga, 2008; Verhaak et al., 2010; Brennan et al., 2013; Wang et al., 2016).

Several clinical features have been associated with the four subtypes. In particular, an association between proneural subtype and age as well as between this subtype and a trend for a longer survival. However, GBM belonging to the proneural subtype have not shown a survival advantage from aggressive treatment protocols. On the other hand, a clear treatment effect has been observed among GBM cases belonging to the classical and mesenchymal subtypes (Tcga, 2008; Verhaak et al., 2010; Brennan et al., 2013; Wang et al., 2016).

The proneural-to-mesenchymal transition upon tumor recurrence has been proposed as a mechanism of treatment resistance for GBM to radiotherapy and/or chemotherapy. GBM patients belonging to the mesenchymal subtype have been associated with survival shorter than the other subtypes, particularly when cases with low transcriptional heterogeneity are considered. Although in the context of poor prognosis patients, GBM cases belonging to the mesenchymal subtype have been found to show favorable response to immunotherapy and intensive radiotherapy and chemotherapy (Verhaak et al., 2010; Behnan et al., 2019).

Long non-coding RNAs (LncRNAs) are RNA transcripts longer than 200 base pairs which do not code for proteins. Although the human genome harbors more than 50,000 LncRNA genes, they are still poor characterized. However, LncRNAs have been found to play key roles in various cell activities related to regulation of gene expression, protein synthesis, stemness, immunity (Schlackow et al., 2017). Moreover, LncRNAs have been found to exert relevant roles in pathogenesis and progression of various cancers including GBM. In particular, a large number of LncRNAs has been found associated with deregulated gene expression and imbalanced biological processes in GBM (Zeng et al., 2018). In this context, the expression of the LncRNA P73 antisense RNA 1T (*TP73-AS1*) has been associated with poor outcome in GBM patients. GBM patients belonging to less aggressive subgroups have been found to be characterized by hypermethylation and low expression of *TP73-AS1*. Moreover, it has been found that *TP73-AS1* downregulation is associated with the loss of aldehyde dehydrogenase 1 family member A1 (*ALDH1A1*) expression and the re-sensitivity of the GBM stem cell (GSC) population to TMZ treatment (Mazor et al., 2019). Expression of the LncRNA HOX transcript antisense intergenic RNA (*HOTAIR*) in GBM has been demonstrated to be significantly higher than in normal tissues and low grade gliomas. Moreover, *HOTAIR* has been demonstrated to be an independent prognostic factor in GBM associated with proliferation and tumorigenic potential of GBM cells (Zhang et al., 2015; Zhang et al., 2018). LncRNA colorectal neoplasia differentially expressed (*CRNDE*) has been found highly expressed in GBM and other brain cancers such as astrocytomas. It has also been explained that its overexpression is associated with promotion of tumor cell growth and migration (Ellis et al., 2014; Wang et al., 2016). LncRNA nuclear enriched abundant transcript 1 (*NEAT1*) has been shown to be a key regulator of nuclear domains implicated in mRNA nuclear retention and splicing. *NEAT1* has been found upregulated in human GBM tissues and GBM cell line models and a high *NEAT1* expression has been associated with larger tumor size, higher WHO grade, higher recurrence rate and unfavorable overall survival (He et al., 2016). The LncRNA X-inactive specific transcript (*XIST*) has been found highly expressed in glioma tissues and GSCc. The knockdown of *XIST* has been shown to suppress proliferation, migration, invasion and tumorigenic potential of GSCs by upregulating *miR152* (Yao et al., 2015). The LncRNA SOX2 overlapping transcript (*SOX2OT*) is characterized by the fact that its transcription genomic region includes the *SOX2* gene; a *SOX2OT* involvement in the transcriptional regulation of *SOX2* has also been observed. *SOX2OT* has been shown to be involved in the proliferation, migration, invasion of GSCs (Su et al., 2017). The LncRNA *H19* has been shown to be upregulated in glioma tissues and associated with poor outcome. Moreover, invasion, angiogenesis, stemness and tumorigenicity of GBM cells have been found enhanced when *H19* is overexpressed (Jiang et al., 2016). The LncRNA *LOC441204* has been found highly expressed in glioma tumor specimens and cell lines. Tumor cell proliferation has been found suppressed by knockdown of *LOC441204* in glioma. On the other hand, *LOC441204*-induced tumor cell growth has been shown to be modulated by the stabilization of the β-catenin pathway (Lin et al., 2017). Regarding the role of other LncRNAs, evidence has also been reported about the fact that the high expression of other LncRNAs such as maternally expressed gene 3 (*MEG3*), metastasis associated lung adenocarcinoma (*MALAT1*), cancer susceptibility candidate 2 (*CASC2*), taurine-upregulated gene 1 (*TUG1*), DBH antisense RNA 1 (*DBH-AS1), AC005035.1*, *AC010336.2*, *AC108134.2*, *AC116351.2*, *Clorf132*, *C10orf91*, *LINC00475*, *MIR210HG* could be associated with poor outcome in GBM cases (Zeng et al., 2018).

## Role of the GBM Tumor Microenvironment

The brain is distinguished from the other organs by the presence of the blood-brain-barrier (BBB). The BBB provides a selective barrier between the systemic circulation and the brain, thus representing a limit for the delivery of many therapeutic agents (Chen et al., 2012; Miura et al., 2013). However, a loss of BBB integrity could be displayed in the presence of cancer, in particular during the cancer progression. This seems to be the reason why several agents, including ICIs, that are known to be not capable of penetrating the BBB, have however shown in some extent a clinical efficacy (de Vries et al., 2006; van Tellingen et al., 2015). Specialized endothelial cells, pericytes, and astrocytic foot processes, dictating junctional integrity, are the elements that constitute the BBB. Moreover, BBB integrity can be also regulated by microglia, being these cells capable of repairing the BBB in a purinergic receptor P2RY12-dependent manner in case of injury (de Vries et al., 2006; van Tellingen et al., 2015).

The complex crosstalk of TME components is involved in the regulation of tumor progression (Quail and Joyce, 2013, 2017; Gritsenko et al., 2012; Nakasone et al., 2012; Quail and Joyce, 2013, 2017). The composition of ECM of normal brain is distinctive, with specific tissue-resident cell types such as neurons, astrocytes and microglia. Moreover, the BBB physically protects the ECM from inflammation (Novak and Kaye, 2000; Mahesparan et al., 2003). The most common component of the brain ECM is hyaluronic acid which is localized in the intraparenchymal region (Kim et al., 2018). The haptotactic cues from the vascular basement membrane, the enrichment of vascular derived chemoctatic cues, as well as interconnected axon tracts can determine the therapeutic resistance of GBM cells in the perivascular space further providing haptotactic cues for cellular invasion (Giese and Westphal, 1996; Nimsky et al., 2005; Gritsenko et al., 2012).

A diffuse invasion pattern characterizes GBM (Young et al., 2015). Healthy tissue beyond the tumor margin is infiltrated by the tumor cells, generally enriched in the GSC stem cell fraction, that either migrate individually or collectively practically impeding complete surgical resection (Eyler and Rich, 2008; Sherriff et al., 2013). On the other hand, GBM tumors rarely intravasate and metastasize from the brain to distant organs (Quail and Joyce, 2013, 2017).

Glioblastoma frequently develop in a hypoxic microenvironment which can modify the metabolic pathways of GBM cells. The brain has a high metabolism level in which the glucose is the major energy substrate and lactate, ketone bodies, fatty acids and aminoacids can also be employed. The metabolic homeostasis of the brain is maintained by the interaction among its various constituent cells such as astrocytes, neurons and microglia (Gritsenko et al., 2012; Nakasone et al., 2012; Quail and Joyce, 2013, 2017). This equilibrium can be altered by genomic aberrations and biochemical variations in GBM cells that often metabolize glucose into lactate even when oxygen is present in a process called Warburg effect. GBM cells can also increase intracellular lipid, aminoacid and nucleotide levels. These metabolic adaptations can favor GBM tumor growth (Gritsenko et al., 2012; Nakasone et al., 2012; Quail and Joyce, 2013, 2017).

Hypervascularity is a characteristic of GBM tumors with an increment in angiogenesis compared to healthy brain tissue. This tumor-associated vasculature is not completely formed, with leaky vessels, and associated with an increase in interstitial fluid pressure. A necrotic core softer than the surrounding tissue characterizes the TME of GBM (Brat and Van Meir, 2004; Brat et al., 2004; Persano et al., 2011; Hambardzumyan and Bergers, 2015; Chen and Hambardzumyan, 2018). High density regions called pseudopalisades are formed when cells migrate away from the hypoxic regions. Increased matrix production with respect to both necrotic regions and healthy tissues characterizes these regions (Brat and Van Meir, 2004; Brat et al., 2004; Persano et al., 2011; Hambardzumyan and Bergers, 2015; Chen and Hambardzumyan, 2018). GBM cells are capable of rapidly invading vasculature (Akiyama et al., 2001; Ponta et al., 2003; Zimmermann and Dours-Zimmermann, 2008; Dicker et al., 2014; Schiffer et al., 2018).

Circulatory and immune systems are connected by the lymphatic system that is involved, together with blood vessels, in the exchange of various elements including fluid, waste, debris as well as immune cells (Engelhardt et al., 2017). Together with the absence of a classic lymphatic drainage system, the CNS exhibits several other peculiar features, such as the presence of tight junctions in the BBB, as well as the limited rejection of foreign tissues within the CNS (Louveau et al., 2015; Schiffer et al., 2018).

**FIGURE 1:**
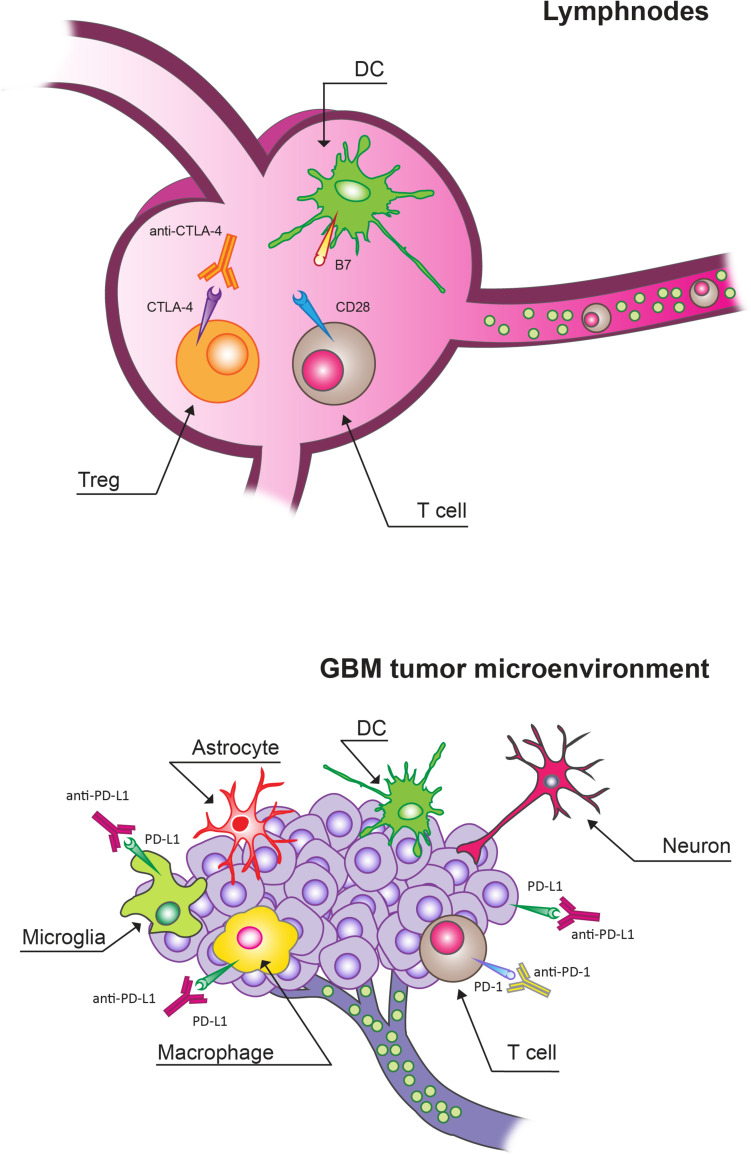
Immune checkpoint inhibitors (ICIs) targets in lymphnodes and in glioblastoma (GBM) tumor microenvironment (TME). Cytotoxic T lymphocyte protein 4 (CTLA-4) blockade mainly acts by targeting Tregs expressing CTLA-4 in lymphnodes. In the context of the GBM TME, programmed cell death protein receptor 1 (PD-1)/programmed death ligand (PD-L1) blockade can overcome the T cell exhaustion and reverse immunosuppression.

There are functional lymphatic vessels in the CNS with the presence of different types of antigen-presenting cells (APCs), including microglia, macrophages, astrocytes and canonical APC such as dendritic cells (DCs; Figure 1; Louveau et al., 2015; Schiffer et al., 2018). In the brain, microglia are the predominant APCs whereas DCs carry out a less relevant role (Lowe et al., 1989; Ulvestad et al., 1994; Weiss et al., 2009; Goldmann et al., 2016).

Activated T cells can invade the CNS. These activated T cells can cover these compartments in an unrestricted manner. On the other hand, antigens can be presented locally or in the draining cervical lymph nodes. Moreover, the BBB can be compromised, thus allowing a relevant infiltration of multiple immune cell types from the peripheral circulation (Weiss et al., 2009). However, GBM tumors present low numbers of tumor-infiltrating lymphocytes (TILs) and other immune effector cell types compared to other tumor types (Schiffer et al., 2018). The interaction of TILs with the TME can cause their re-education. In particular, the local TME can alter T cell effector function in the process related to anti-tumor immunity even in the CNS, where T cell-mediated inflammatory responses are considered poor under normal physiological contexts. The number of antigen-specific TILs can remain relatively low besides frequently displaying an exhausted phenotype. The peculiar immune environment of the brain can be responsible for this reduced quantity and limited activity of T cells in GBM. In particular, there is a specific need of avoiding unrestrained inflammation in the brain given its solid enclosure and the potential for damage from increased intracranial pressure (Quail and Joyce, 2013, 2017; Gajewski et al., 2017; Keskin et al., 2019). This need is not present with the same extent in peripheral organs. In fact, this environment in which both inflammatory and adaptive immune responses are tightly regulated is specific of the brain; besides there is a variety of immunosuppressive mechanisms at both the molecular and cellular levels (Perng and Lim, 2015). In particular, stromal cells of the brain produce high levels of the classic immunosuppressive cytokines transforming growth factor β (TGFβ), interleukin-10 (IL-10) in response to inflammatory stimuli, including those derived from GBM tumors, in order to maintain homeostasis (Vitkovic et al., 2001; Gong et al., 2012). Furthermore, the accumulation of regulatory T cells (Tregs) is stimulated by IDO which can suppress T cell activity by depleting tryptophan from the microenvironment. Microglia and tumor- infiltrating myeloid cells can also inhibit T cell proliferation and function through the production of high levels of arginase that causes the depletion of tissue arginine levels (Uyttenhove et al., 2003; Fecci et al., 2006a, b; Wainwright et al., 2012).

Immune checkpoints exert a key role in central and peripheral tolerance by counteracting activating signaling (Xu et al., 2018). Under physiological conditions, immune checkpoint molecules represent a negative feedback to regulate inflammatory responses following T cell activation (Krummel and Allison, 1996; Chambers et al., 2001; Collins et al., 2002; Stone et al., 2009; Inarrairaegui et al., 2018). A mechanism used by tumors, including GBM, to inhibit and escape the anti-tumor immune response is represented by the expression of checkpoint molecules, such as cytotoxic T-lymphocyte antigen 4 (CTLA-4) and PD1 (Stone et al., 2009; Francisco et al., 2010; Cheng et al., 2013; Bhandaru and Rotte, 2017; Hui et al., 2017; Wei et al., 2017, 2018, 2019a,b; Rotte et al., 2018; Kalbasi and Ribas, 2020; Sharma and Allison, 2020).

## Small Molecules for Targeted Therapies in GBM

**FIGURE 2:**
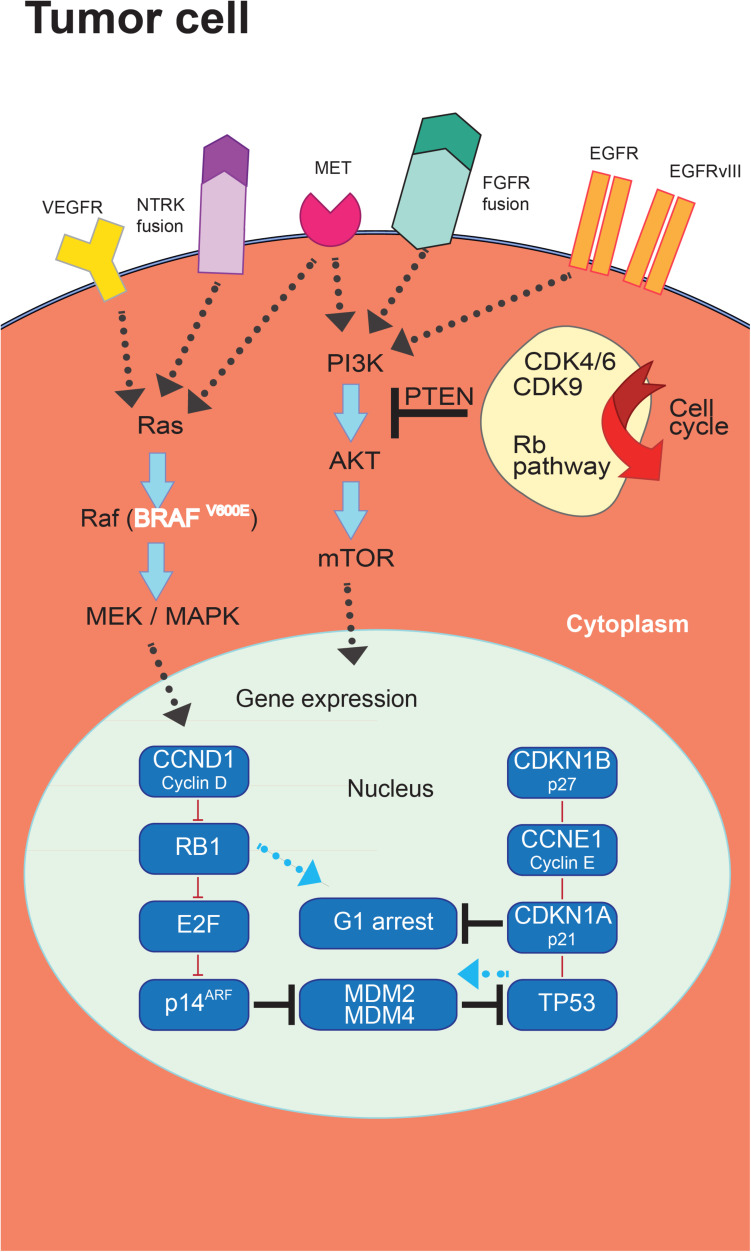
Targeted therapies in glioblastoma (GBM). The introduction of novel targeted therapies has been allowed by the comprehensive characterization of the molecular landscape of somatic genomic alterations identifying a series of mutated genes and abnormal rearrangements potentially utilizable as therapeutic targets.

The progresses in the molecular classification of GBM have allowed the identification of dysregulated pathways that could represent potential targets for new treatment strategies (Figure 2).

Glioblastoma is a vascularized tumor which is histologically characterized by the expression of VEGF and other proangiogenic cytokines involved in the stimulation of endothelial cell proliferation, migration and survival (Schiffer et al., 2018). In patients with a relapsed GBM, the TKI regorafenib has received approval in the GBM treatment (Lombardi et al., 2019).

Other TKIs targeting VEGF family components have been proposed for the treatment of GBM besides regorafenib. Of note, vascular normalization has been proposed as an alternative strategy for the employment of antiangiogenic therapies in which the objective is to modulate the tumor vasculature in order to reduce hypoxia, and to support physiological angiogenesis. This process could ultimately improve perfusion and drug delivery. In this context, promising results in reducing angiogenesis and normalizing vascularization have been shown by cediranib and sutinib (Batchelor et al., 2013; Grisanti et al., 2019).

The PI3K/mammalian target of rapamycin (mTOR) pathway is a targetable pathway in GBM. In this context, the mTOR inhibitor temsirolimus did not show a treatment efficacy as single agent in recurrent GBM (Chang et al., 2005). Similarly, the pan-PI3K inhibitor buparlisib did not demonstrate a treatment efficacy (Wen et al., 2019). Also treatment combinations of mTOR pathway inhibitors with radiotherapy and TMZ or in combination with radiotherapy only did not show efficacy (Ma et al., 2015; Wick et al., 2016).

Targeting MDM2 and mouse double minute 4 homolog (MDM4) activity has been suggested for GBM cases carrying *MDM2* or *MDM4* gene amplification (Wick et al., 2019).

Moreover, the *CDK4/6* inhibitor palbociclib failed to demonstrate the efficacy of this treatment in GBM (Taylor et al., 2018). CDK9 is an alternative targetable CDK (Taylor et al., 2018).

The use of TKIs targeting EGFR as single agents did not demonstrate significant activity for GBM treatment (Lassman et al., 2005; Hegi et al., 2011). It has not yet been agreed on the potential use of MET as target for GBM treatment. The use of the TKIs crizotinib and cabozantinib in recurrent GBM has achieved modest efficacy after several attempts (International Cancer Genome Consortium PedBrain Tumor Project, 2016; Wen et al., 2018). Tests have been carried out for larotrectinib and entrectinib in neutrophic tyrosine receptor kinase (NTRK) fusion-positive GBM without any confirmation on their efficacy (Ferguson et al., 2018). Notwithstanding the frequent expression in GBM of fibroblast growth factor receptors (FGFRs), a relevance as potential therapy target seems to be restricted to GBM exhibiting FGFR-transforming acidic coiled-coil containing protein TACC fusions (Singh et al., 2012), as shown by using the pan-FGFR kinase inhibitor erdafitinib (Di Stefano et al., 2015). A modest treatment efficacy has been obtained for the possible targeting of *BRAF*V600E mutation in GBM (Kaley et al., 2018). Finally, eribulin has been proposed to inhibit TERT activity in GBM (Takahashi et al., 2019). A list of the current clinical trials employing TKIs for GBM treatment is reported in Table 1.

**TABLE 1:** Clinical trials in glioblastoma (GBM) using tyrosine kinase inhibitors (TKIs).

Title	Condition or Disease	Intervention/Treatment	NCT Number	Status	Status Phase
mTORC1/mTORC2 kinase inhibitor AZD2014 in previously treated glioblastoma multiforme	Glioblastoma multiforme	Drug: AZD2014	NCT02619864	Completed	Phase I
Gefitinib and radiation therapy in treating patients with glioblastoma multiforme	Adult giant cell Glioblastoma Adult glioblastoma Adult gliosarcoma	Drug: Gefitinib	NCT00052208	Completed	Phase I, II
Study of AEE788 in patients with recurrent/relapse glioblastoma multiforme (GBM)	Glioblastoma multiforme	Drug: AEE788	NCT00116376	Completed	Phase I, II
Clinical trial on the combination of avelumab and axitinib for the treatment of patients with recurrent glioblastoma	Recurrent glioblastoma (WHO-grade IV glioma)	Drug: Axitinib Drug: Avelumab	NCT03291314	Completed	Phase II
AZD8055 for adults with recurrent gliomas	Glioblastoma Multiforme Anaplastic astrocytoma Anaplastic oligodendroglioma Malignant glioma Brainstem glioma	Drug: AZD8055	NCT01316809	Completed	Phase I
Sunitinib in treating patients with recurrent malignant gliomas	Astrocytoma Adult diffuse astrocytoma Adult giant cell Glioblastoma Adult glioblastoma and other 5	Drug: Sunitinib malate	NCT00499473	Completed	Phase II
Study to assess safety, pharmacokinetics, and efficacy of oral CC-223 for patients with advanced solid tumors, non-hodgkin lymphoma or multiple myeloma	Multiple myeloma Diffuse large B cell Lymphoma Glioblastoma multiforme Hepatocellular carcinoma and other 4	Drug: CC-223	NCT01177397	Completed	Phase I, II
Trial of ponatinib in patients with bevacizumab-refractory glioblastoma	Glioblastoma	Drug: Ponatinib	NCT02478164	completed	Phase II
A phase II exploratory, multicentre, open-label, non-comparative study of ZD1839 (iressa) and radiotherapy in the treatment of patients with glioblastoma multiforme	Glioblastoma	Drug: Gefitinib	NCT00238797	Completed	Phase II
A study of the safety and efficacy of tarceva in patients with first relapse of grade IV glioma (glioblastoma multiforme)	Glioblastoma	Drug: Erlotinib HCl (OSI-774)	NCT00337883	Completed	Phase II
Study of tesevatinib monotherapy in patients with recurrent glioblastoma	Glioblastoma Recurrent glioblastoma Brain tumor	Drug: Tesevatinib	NCT02844439	Completed	Phase II
Oral tarceva study for recurrent/residual glioblastoma multiforme and anaplastic astrocytoma	Glioblastoma multiforme Anaplastic astrocytoma	Drug: Erlotinib	NCT00301418	Completed	Phase I, II
Gefitinib in treating patients with newly diagnosed glioblastoma multiforme	Adult giant cell glioblastoma Adult glioblastoma Adult gliosarcoma	Drug: Gefitinib	NCT00014170	Completed	Phase II
Erlotinib and sorafenib in treating patients with progressive or recurrent glioblastoma multiforme	Adult giant cell glioblastoma Adult glioblastoma Adult gliosarcoma Recurrent adult brain tumor	Drug: erlotinib hydrochloride Drug: sorafenib tosylate	NCT00445588	Completed	Phase II
AZD2171 in treating patients with recurrent glioblastoma multiforme	Adult giant cell Glioblastoma Adult glioblastoma Adult gliosarcoma Recurrent adult brain tumor	Drug: cediranib maleate	NCT00305656	Completed	Phase II
Dasatinib in treating patients with recurrent glioblastoma multiforme or gliosarcoma	Adult giant cell glioblastoma Adult glioblastoma Adult gliosarcoma Recurrent adult brain neoplasm	Drug: Dasatinib	NCT00423735	Completed	Phase II
Cediranib maleate and cilengitide in treating patients with progressive or recurrent glioblastoma	Adult giant cell Glioblastoma Adult glioblastoma Adult gliosarcoma Recurrent adult brain neoplasm	Drug: Cediranib maleate Drug: Cilengitide	NCT00979862	Completed	Phase I
Sorafenib in newly diagnosed high grade glioma	Glioblastoma Gliosarcoma Anaplastic astrocytoma Anaplastic oligoastrocytoma Anaplastic oligodendroglioma	Drug: Sorafenib dose escalation	NCT00884416	Completed	Phase I
E7050 in combination with E7080 in subjects with advanced solid tumors (dose escalation) and in subjects with recurrent glioblastoma or unresectable stage III or stage IV melanoma after prior systemic therapy (expansion cohort and phase 2)	Advanced solid tumors	Drug: E7050 Drug: Lenvatinib	NCT01433991	Completed	Phase I, II
ZD 1839 in treating patients with glioblastoma multiforme in first relapse	Brain and central nervous system tumors	Drug: Gefitinib	NCT00016991	Completed	Phase II
Ph I dasatinib + erlotinib in recurrent MG	Glioblastoma, Gliosarcoma	Drug: Erlotinib and dasatinib	NCT00609999	Completed	Phase I
Bafetinib in treating patients with recurrent high-grade glioma or brain metastases	Adult anaplastic astrocytoma Adult anaplastic Ependymoma Adult anaplastic Oligodendroglioma Adult giant cell glioblastoma Adult glioblastoma and other 5	Drug: Bafetinib	NCT01234740	Completed	Phase I
A randomized phase II clinical trial on the efficacy of axitinib as a monotherapy or in combination with lomustine for the treatment of patients with recurrent glioblastoma	Glioblastoma multiforme	Drug: Axitinib Drug: Axitinib plus lomustine	NCT01562197	Completed	Phase II
Cediranib in combination with lomustine chemotherapy in recurrent glioblastoma	Recurrent glioblastoma	Drug: Cediranib Drug: Lomustine chemotherapy Drug: Placebo Cediranib	NCT00777153	Completed	Phase III
Radiation therapy and temozolomide followed by temozolomide plus sorafenib for glioblastoma multiforme	Glioblastoma multiforme	Drug: Temozolomide Drug: Sorafenib	NCT00544817	Completed	Phase II
PTK787/ZK 222584 in combination with temozolomide and radiation in patients with glioblastoma taking enzyme-inducing anti-epileptic drugs	Glioblastoma	Drug: PTK787/ZK 222584 Drug: Temozolomide	NCT00385853	Completed	Phase I
Study of imatinib mesylate in combination with hydroxyurea versus hydroxyurea alone as an oral therapy in patients with temozolomide resistant progressive glioblastoma	Glioblastoma multiforme Astrocytoma	Drug: Imatinib mesylate Drug: Hydroxyurea	NCT00154375	Completed	Phase III
Open label trial to explore safety of combining afatinib (BIBW 2992) and radiotherapy with or without temozolomide in newly diagnosed glioblastoma multiform	Glioblastoma	Drug: Temozolomide Drug: BIBW2992	NCT00977431	Completed	Phase I
Ph. 2 sorafenib + protracted temozolomide in recurrent GBM	Recurrent glioblastoma Multiforme	Drug: Sorafenib and temozolomide	NCT00597493	Completed	Phase II
Erlotinib and temozolomide with radiation therapy in treating patients with glioblastoma multiforme or other brain tumors	Adult giant cell glioblastoma Adult glioblastoma Adult gliosarcoma	Drug: Erlotinib hydrochloride Drug: Temozolomide	NCT00039494	Completed	Phase II
Erlotinib and radiation therapy in treating young patients with newly diagnosed glioma	Brain and central nervous system tumors	Drug: Erlotinib hydrochloride	NCT00124657	Completed	Phase I, II
Safety and efficacy study of tarceva, temodar, and radiation therapy in patients with newly diagnosed brain tumors	Glioblastoma multiforme Gliosarcoma	Drug: Tarceva Drug: Temodar	NCT00187486	Completed	Phase II
Study of sunitinib before and during radiotherapy in newly diagnosed biopsy-only glioblastoma patients	Glioblastoma	Drug: Sunitinib	NCT01100177	Completed	Phase II
Dasatinib and bevacizumab in treating patients with recurrent or progressive high-grade glioma or glioblastoma multiforme	Glioblastoma multiforme	Biological: Bevacizumab Drug: Basatinib	NCT00892177	Completed	Phase II
Lapatinib in treating patients with recurrent glioblastoma multiforme	Brain and central nervous system tumors	Drug: Lapatinib ditosylate	NCT00099060	Completed	Phase I, II
Gefitinib in treating patients with recurrent or progressive CNS tumors	Brain and central nervous system tumors	Drug: Gefitinib	NCT00025675	Completed	Phase II
Radiation therapy, temozolomide, and erlotinib in treating patients with newly diagnosed glioblastoma multiforme	CNS tumor Adult	Drug: Erlotinib hydrochloride Drug: Temozolomide	NCT00274833	Completed	Phase II
Sunitinib tumor levels in patients not on enzyme-inducing anti-epileptic drugs undergoing debulking surgery for recurrent glioblastoma	Glioblastoma Brain tumor	Drug: Sunitinib	NCT00864864	Completed	Early Phase I
Study of bevacizumab plus temodar and tarceva in patients with glioblastoma or gliosarcoma	Glioblastoma Gliosarcoma	Drug: Bevacizumab Drug: Tarceva Drug: Temozolomide	NCT00525525	Completed	Phase II
A Phase II trial of sutent (sunitinib; SU011248) for recurrent anaplastic astrocytoma and glioblastoma	Anaplastic astrocytoma Glioblastoma	Drug: Sunitinib malate	NCT00606008	Completed	Phase II
Cediranib, temozolomide, and radiation therapy in treating patients with newly diagnosed glioblastoma	Adult giant cell Glioblastoma Adult glioblastoma Adult gliosarcoma	Drug: Cediranib maleate Drug: Temozolomide	NCT00662506	Completed	Phase I, II
Dasatinib or placebo, radiation therapy, and temozolomide in treating patients with newly diagnosed glioblastoma multiforme	Brain and central nervous system tumors	Drug: Dasatinib Drug: Temozolomide	NCT00869401	Completed	Phase I, II
Erlotinib compared with temozolomide or carmustine in treating patients with recurrent glioblastoma multiforme	Brain and central nervous system tumors	Drug: Carmustine Drug: Erlotinib hydrochloride Drug: Temozolomide	NCT00086879	Completed	Phase II
Sorafenib combined with erlotinib, tipifarnib, or temsirolimus in treating patients with recurrent glioblastoma multiforme or gliosarcoma	Adult giant cell glioblastoma Adult glioblastoma Adult gliosarcoma Recurrent adult brain tumor	Drug: Sorafenib tosylate Drug: Erlotinib hydrochloride Drug: Tipifarnib Drug: Temsirolimus	NCT00335764	Completed	Phase I, II
Phase II Imatinib + hydroxyurea in treatment of patients with recurrent/progressive grade II low-grade glioma (LGG)	Glioblastoma Gliosarcoma	Drug: Imatinib mesylate and hydroxyurea	NCT00615927	Completed	Phase II
Sorafenib tosylate and temsirolimus in treating patients with recurrent glioblastoma	Adult glioblastoma Adult gliosarcoma Recurrent adult brain neoplasm	Drug: Sorafenib tosylate Drug: Temsirolimus	NCT00329719	Completed	Phase I, II
Phase I : cediranib in combination with lomustine chemotherapy in recurrent malignant brain tumor	Recurrent glioblastoma Brain tumor	Drug: Cediranib Drug: Lomustine	NCT00503204	Completed	Phase I
Ph I SU011248 + irinotecan in treatment of Pts w MG	Glioblastoma	Drug: SU011248 and irinotecan	NCT00611728	Completed	Phase I
Ph I zactima + imatinib mesylate and hydroxyurea for pts w recurrent malignant glioma	Glioblastoma Gliosarcoma	Drug: Zactima, gleevec, hydroxyurea	NCT00613054	Completed	Phase I
Imatinib mesylate and hydroxyurea in treating patients with recurrent or progressive meningioma	Glioblastoma Gliosarcoma	Drug: Hydroxyurea Drug: Imatinib mesylate	NCT00354913	Completed	Phase II
Bevacizumab and sorafenib in treating patients with recurrent glioblastoma multiforme	Brain and central nervous system tumors	Biological: Bevacizumab Drug: Sorafenib tosylate	NCT00621686	Completed	Phase II
BIBW 2992 (afatinib) with or without daily temozolomide in the treatment of patients with recurrent malignant glioma	Glioma	Drug: BIBW 2992 Drug: TMZ Drug: BIBW 2992 plus TMZ	NCT00727506	Completed	Phase II
Bevacizumab and erlotinib after radiation therapy and temozolomide in treating patients with newly diagnosed glioblastoma multiforme or gliosarcoma	Brain and central nervous system tumors	Drug: Bevacizumab Drug: Erlotinib hydrochloride	NCT00720356	Completed	Phase II
Ph I gleevec in combo w RAD001 + hydroxyurea for Pts w recurrent MG	Glioblastoma Gliosarcoma	Drug: Gleevec, RAD001, and hydroxyurea	NCT00613132	Completed	Phase I
GW572016 to treat recurrent malignant brain tumors	Glioma Brain tumor Glioblastoma multiforme GBM Gliosarcoma GS	Drug: Lapatinib ditosylate	NCT00107003	Completed	Phase II
Temozolomide and radiation therapy with or without vatalanib in treating patients with newly diagnosed glioblastoma multiforme	Brain and central nervous system tumors	Drug: Temozolomide Drug: Vatalanib	NCT00128700	Completed	Phase I, II
Ph II erlotinib + sirolimus for pts w recurrent malignant glioma multiforme	Glioblastoma Gliosarcoma	Drug: Erlotinib + Sirolimus	NCT00672243	Completed	Phase II
Afatinib (BIBW 2992) QTcF trial in patients with relapsed or refractory solid tumors	Neoplasms	Drug: BIBW 2992	NCT00875433	Completed	Phase II
Phase (Ph) II bevacizumab + erlotinib for patients (Pts) with recurrent malignant glioma (MG)	Glioblastoma Gliosarcoma	Drug: Bevacizumab and erlotinib	NCT00671970	Completed	Phase II
Everolimus and gefitinib in treating patients with progressive glioblastoma multiforme or progressive metastatic prostate cancer	Brain and central nervous system tumors Prostate cancer	Drug: Everolimus Drug: Gfitinib	NCT00085566	Completed	Phase I, II
Sorafenib in treating patients with recurrent or progressive malignant glioma	Adult anaplastic astrocytoma Adult anaplastic Oligodendroglioma Adult giant cell Glioblastoma and other 2	Drug: Sorafenib tosylate	NCT00093613	Completed	Phase I
AZD7451 for recurrent gliomas	Glioblastoma multiforme	Drug: AZD7451	NCT01468324	Completed	Phase I
Gefitinib and radiation therapy in treating children with newly diagnosed gliomas	Untreated childhood anaplastic astrocytoma Untreated childhood anaplastic oligodendroglioma Untreated childhood brain stem glioma Untreated childhood giant cell glioblastoma and other 4	Drug: Gefitinib	NCT00042991	Completed	Phase I, II
Erlotinib in treating patients with recurrent malignant glioma or recurrent or progressive meningioma	Adult anaplastic astrocytoma Adult anaplastic oligodendroglioma Adult giant cell glioblastoma Adult glioblastoma and other 5	Drug: Erlotinib hydrochloride	NCT00045110	Completed	Phase I, II
Gamma-secretase inhibitor RO4929097 and cediranib maleate in treating patients with advanced solid tumors	Adult anaplastic astrocytoma Adult anaplastic ependymoma Adult anaplastic oligodendroglioma Adult brain stem glioma Adult giant cell glioblastoma Adult glioblastoma and other 41	Secretase inhibitor RO4929097 Drug: Cediranib maleate	NCT01131234	Completed	Phase I
EGFR inhibition using weekly erlotinib for recurrent malignant gliomas	Brain cancer	Drug: Erlotinib	NCT01257594	Completed	Phase I
Lapatinib in treating young patients with recurrent or refractory central nervous system tumors	Recurrent childhood anaplastic Astrocytoma Recurrent childhood brain stem gliom Recurrent childhood ependymoma Recurrent childhood giant cell glioblastoma Recurrent childhood glioblastoma and other 3	Drug: Lapatinib ditosylate	NCT00095940	Completed	Phase I, II
Erlotinib in treating patients with solid tumors and liver or kidney dysfunction	Astrocytoma Adult anaplastic ependymoma Adult anaplastic oligodendroglioma				
Adult brain stem glioma Adult diffuse astrocytoma Adult ependymoblastoma Adult giant cell glioblastoma and 79 more	Drug: Erlotinib hydrochloride	NCT00030498	Completed	Phase I
ZD1839 and oral irinotecan in treating young patients with refractory solid tumors	Glioblastoma Rhabdomyosarcomas Neuroblastoma Osteosarcoma	Drug: Irinotecan, Gefitinib	NCT00132158	Completed	Phase I
Apatinib in recurrent or refractory intracranial central nervous system malignant tumors	Efficacy and safety	Drug: Apatinib Drug: Temodar	NCT03660761	Completed	Phase II
Bevacizumab and cediranib maleate in treating patients with metastatic or unresectable solid tumor, lymphoma, intracranial glioblastoma, gliosarcoma, or anaplastic astrocytoma	Adult grade III lymphomatoid granulomatosis Adult nasal type extranodal NK/T-cell lymphoma Anaplastic large cell lymphoma Angioimmunoblastic T-cell lymphoma	Biological: Bevacizumab Drug: Cediranib maleate	NCT00458731	Completed	Phase I
	Childhood burkitt lymphoma and other 56				
Erlotinib and Temsirolimus in Treating Patients With Recurrent Malignant Glioma	Adult anaplastic astrocytoma Adult anaplastic oligodendroglioma Adult diffuse astrocytoma Adult giant cell glioblastoma Adult glioblastoma and other 6	Drug: Erlotinib Drug: Temsirolimus	NCT00112736	Completed	Phase I, II
Pazopanib in combination with lapatinib in adult patients with relapsed malignant glioma (VEG102857)	Glioma	Drug: Pazopanib Drug: Lapatinib	NCT00350727	Completed	Phase I
BIBF 1120 for recurrent high-grade gliomas	Glioblastoma Gliosarcoma Anaplastic astrocytoma Anaplastic oligodendroglioma Anaplastic oligoastrocytoma	Drug: BIBF 1120	NCT01380782	Completed	Phase II
Imetelstat sodium in treating young patients with refractory or recurrent solid tumors or lymphoma	Brain and central nervous system tumors Lymphoma Lymphoproliferative disorder Small intestine cancer Unspecified childhood solid tumor, protocol specific	Drug: Imetelstat sodium	NCT01273090	Completed	Phase I
Imatinib mesylate in treating patients with gliomas	Brain and central nervous system tumors	Drug: Imatinib mesylate	NCT00039364	Completed	Phase II
Imatinib mesylate in treating patients with recurrent malignant glioma or meningioma	Brain and central nervous system tumors	Drug: Imatinib mesylate	NCT00010049	Completed	Phase I, II
Tumor tissue analysis in patients receiving imatinib mesylate for malignant glioma	Brain and central nervous system tumors	Drug: Imatinib mesylate	NCT00401024	Completed	Phase I
Imatinib mesylate, vatalanib, and hydroxyurea in treating patients with recurrent or relapsed malignant glioma	Brain and central nervous system tumors	Drug: Hydroxyurea Drug: Imatinib mesylate Drug: Vatalanib	NCT00387933	Completed	Phase I
Gefitinib plus temozolomide in treating patients with malignant primary glioma	Brain and central nervous system tumors	Drug: Gefitinib Drug: Temozolomide	NCT00027625	Completed	Phase I
Imatinib mesylate and temozolomide in treating patients with malignant glioma	Brain and central nervous system tumors	Drug: Imatinib mesylate Drug: Temozolomide	NCT00354068	Completed	Phase I
Erlotinib and sirolimus in treating patients with Recurrent malignant glioma	Brain and central nervous system tumors	Drug: Erlotinib + Sirolimus	NCT00509431	Completed	Phase I
SU5416 in treating patients with recurrent astrocytoma or mixed glioma that has not responded to radiation therapy	Brain and central nervous system tumors	Drug: Semaxanib	NCT00004868	Completed	Phase I, II
Lenalidomide in combination with bevacizumab, sorafenib, temsirolimus, or 5-fluorouracil, leucovorin, oxaliplatin (FOLFOX)	Advanced cancers	Drug: Lenalidomide Drug: Bevacizumab Drug: Sorafenib Drug: Temsirolimus Drug: Oxaliplatin Drug: Leucovorin Drug: 5-fluorouracil	NCT01183663	Completed	Phase I

*Summarized in the Table 1 are the ongoing clinical trials present on ClinicalTrials.gov searching the keywords “glioblastoma multiforme” and “kinase inhibitor” The research has been done adding the following filters: “Completed”; “The research has been performed on October 21st, 2020.”*

## Use of Icis for GBM Treatment

Following the results of ICIs use in other cancers, the use of PD-1/PD-L1 inhibitors has been proposed for GBM (Table 2). Clinical trial results have shown that GBM patients with unresectable tumors do not benefit from monotherapy with nivolumab in terms of survival improvement when compared to bevacizumab (Reiss et al., 2017). Moreover, pembrolizumab showed limited activity for GBM (Reardon et al., 2014, 2016; Schwartz et al., 2016; Reardon et al., 2017; Reiss et al., 2017; Wen et al., 2018).

**TABLE 2:** Clinical trials in glioblastoma (GBM) using immune checkpoint inhibitors (ICIs).

Title	Condition or Disease	Intervention/Treatment	NCT Number	Status	Status Phase
Neoantigen-based personalized vaccine combined with immune checkpoint blockade therapy in patients with newly diagnosed, unmethylated glioblastoma	Glioblastoma	Biological: NeoVax Biological: Nivolumab Biological: Ipilimumab	NCT03422094	Suspended	Phase I
Autologous dendritic cells, metronomic cyclophosphamide and checkpoint blockade in children with relapsed HGG	Childhood glioblastoma	Drug: depletion of regulatory T cells Biological: cancer vaccine Biological: checkpoint blockade	NCT03879512	Recluting	Phase I, II
Cytokine microdialysis for real-time immune monitoring in glioblastoma patients undergoing checkpoint blockade	Glioblastoma	Drug: Nivolumab Drug: BMS-986016	NCT03493932	Recluting	Phase I
Laser interstitial thermotherapy (LITT) combined with checkpoint inhibitor for recurrent GBM (RGBM)	Glioblastoma Adult	Drug: Pembrolizumab at 7 days prior Drug: Pembrolizumab at 14 days post Drug: Pembrolizumab at 35 days post	NCT03277638	Recluting	Phase I, II
Pilot surgical trial to evaluate early immunologic pharmacodynamic parameters for the PD-1 checkpoint inhibitor, pembrolizumab (MK-3475), In patients with surgically accessible recurrent/progressive glioblastoma	Brain cancer	Drug: MK-3475	NCT02852655	Active, not recruiting	Phase I
A study testing the effect of immunotherapy (ipilimumab and nivolumab) in patients with recurrent glioblastoma with elevated mutational burden	Recurrent glioblastoma Secondary glioblastoma	Biological: Ipilimumab Biological: Nivolumab	NCT04145115	Not yet recruiting	Phase II
First-in-human, phase 1b/2a trial of a multipeptide therapeutic vaccine in patients with progressive glioblastoma	Glioblastoma Adult	Biological: Multiple dose of EO2401	NCT04116658	Not yet recruiting	Phase I, II
A phase 1 study of PVSRIPO and pembrolizumab in patients with recurrent glioblastoma	Glioblastoma Recurrent glioblastoma Supratentorial glioblastoma Brain tumor	Biological: PVSRIPO Biological: Pembrolizumab	NCT04479241	Not yet recruiting	Phase I
Nivolumab, BMS-986205, and radiation therapy with or without temozolomide in treating patients with newly diagnosed glioblastoma	Glioblastoma	Biological: IDO1 Inhibitor BMS-986205 Biological: Nivolumab Drug: Temozolomide	NCT04047706	Recluting	Phase I
Immunogene-modified T (IgT) cells against glioblastoma multiforme	Glioblastoma multiforme of brain Glioblastoma multiforme	Biological: Antigenspecific IgT cells	NCT03170141	Enrolling by invitation	Phase I
An investigational immunotherapy study of nivolumab compared to temozolomide, each given with radiation therapy, for newly diagnosed patients with glioblastoma (GBM, a malignant brain cancer)	Brain Cancer	Drug: Nivolumab Drug: Temozolomide	NCT02617589	Active, not recruiting	Phase III
Translational study of nivolumab in combination with bevacizumab for recurrent glioblastoma	Recurrent adult brain tumor	Drug: Nivolumab Drug: Bevacizumab	NCT03890952	Recluting	Phase II
Immunological and functional characterization of cellular population CD45+ infiltrating human glioblastoma	Glioblastoma		NCT03687099	Recluting	Observational
Avelumab in patients with newly diagnosed glioblastoma multiforme	Glioblastoma Multiforme of brain	Biological: Avelumab	NCT03047473	Active, not recruiting	Phase II
Capecitabine + bevacizumab in patients with recurrent glioblastoma	Glioblastoma	Drug: Capecitabine Drug: Bevacizumab	NCT02669173	Recluting	Phase I
VXM01 plus avelumab combination study in progressive glioblastoma	Recurrent glioblastoma	Biological: VXM01 Biological: Avelumab	NCT03750071	Recluting	Phase I, II
Immunotherapy (nivolumab and ipilimumab) before and after surgery for the treatment of recurrent or progressive high grade glioma in children and young adults	Glioblastoma Malignant glioma Recurrent glioblastoma Recurrent malignant glioma Recurrent grade III Glioma Grade III GLioma	Biological: Ipilimumab Biological: Nivolumab Drug: Placebo Administratio	NCT04323046	Not yet recruiting	Phase I
CART-EGFRvIII + Pembrolizumab in GBM	Glioblastoma	Biological: CARTEGFRvIII T cells Biological: Pembrolizumab	NCT03726515	Active, not recruiting	Phase I
INO-5401 and INO-9012 delivered by electroporation (EP) in combination with cemiplimab (REGN2810) in newly diagnosed glioblastoma (GBM)	Glioblastoma	Biological: INO-5401 Biological: INO-9012 Biological: Cemiplimab Drug: Temozolomide	NCT03491683	Active, not recruiting	Phase I, II
Combination adenovirus + pembrolizumab to trigger immune virus effects	Brain cancer Brain neoplasm Glioma Glioblastoma Gliosarcoma and other 3	Biological: DNX-2401 Biological: Pembrolizumab	NCT02798406	Active, not recruiting	Phase II
GMCI, nivolumab, and radiation therapy in treating patients with newly diagnosed high-grade gliomas	Glioma Malignant	Biological: AdV-tk Drug: Valacyclovir Drug: Temozolomide Biological: Nivolumab	NCT03576612	Recluting	Phase I
Nivolumab, BMS-986205, and radiation therapy with or without temozolomide in treating patients with newly diagnosed glioblastoma	Glioblastoma	Biological: IDO1 Inhibitor BMS-986205 Biological: Nivolumab Drug: Temozolomide	NCT04047706	Recluting	Phase I
Study of the IDO pathway inhibitor, indoximod, and temozolomide for pediatric patients with progressive primary malignant brain tumors	Glioblastoma Multiforme Glioma Gliosarcoma Malignant brain tumor Ependymoma and other 3	Drug: Indoximod Drug: Temozolomide Drug: Cyclophosphamide Drug: Etoposide	NCT02502708	Active, not recruiting	Phase I
A phase 0 study of AZD1775 in recurrent GBM patients	Glioblastoma	Biological: AZD1775	NCT02207010		Early phase I
Nivolumab in people with IDH-mutant gliomas with and without hypermutator phenotype	Glioma Glioblastoma High grage glioma Low grade glioma Malignant glioma	Drug: Nivolumab	NCT03718767	Recluting	Phase II
A pilot study to evaluate PBR PET in brain tumor patients treated with chemoradiation or immunotherapy	Intracranial tumors Glioblastoma Melanoma	Biological: Cancer immunotherapy Radiation: Radiation and chemotherapy	NCT02431572	Completed	
HSV G207 with a single radiation dose in children with recurrent high-grade glioma	Neoplasms High grade glioma Glioblastoma multiforme Malignant glioma of brain Anaplastic astrocytoma of brain and other 3	Drug: Biological G207	NCT04482933	Not yet recruiting	Phase II

*Summarized in the Table are the ongoing clinical trials present on ClinicalTrials.gov searching the keywords “glioblastoma multiforme” and “Checkpoint.” The research has been done adding the following filters: “Not yet recruiting”; “Recruiting”; “Enrolling by invitation”; “Active, not recruiting”; “Suspended”; “Terminate”; “Completed”; “Withdrawn”; “Unknown status”; “The research has been performed on October 21st, 2020.”*

Recent tests have been carried out involving patients with newly diagnosed or relapsed GBMs for the use of ICIs (e.g., nivolumab or pembrolizumab) in neoadjuvant and/or adjuvant administration, although no straightforward results have been obtained (Cloughesy et al., 2019; Schalper et al., 2019).

Glioblastoma tumors of cases non-responsive to ICIs have shown an enrichment in mutations of the *PTEN* gene (Zhao et al., 2019) that has been associated with an immunosuppressive TME characterized by the presence of GBM cells expressing CD44. *PTEN* mutant tumors were characterized by highly clustering tumor cells with a lack of T cell infiltration (Peng et al., 2016; George et al., 2017). Furthermore, the poor responsiveness to ICIs of GBM cases carrying *PTEN* mutations has been related to a low PD-L1 expression for the involvement of the PI3K-mTOR pathway that is downstream to PTEN (Lastwika et al., 2016).

Responsiveness to ICI was associated with the presence of mutations of *BRAF*/protein tyrosine phosphatase non-receptor type 11 (*PTPN11*). In this subset of *BRAF*/*PTPN11* GBM patients, treatment combinations of ICIs and MAPK inhibitors could be introduced (Toso et al., 2014; Toso et al., 2014; Ebert et al., 2016; Wang et al., 2016).

The heterogeneous response rate to ICIs highlights the need of identifying the subgroups of patients who could benefit the most from the use of this immunotherapy treatment. PD-L1 expression was the first marker evaluated as predictor of a clinical response to ICIs (Ansell et al., 2015). PD-L1 expression in gliomas was associated with *IDH* status (Berghoff and Preusser, 2016; Garber et al., 2016; Berghoff et al., 2017). Importantly, mesenchymal GBM has been found having high levels of PD-L1 expression that may suggest that the expression of immune checkpoint proteins and aggressiveness of GBM tumors may be correlated (Garber et al., 2016). More recently, the tumor mutational burden has been proposed as a predictive marker of responsiveness to ICIs. However, it has not generally been demonstrated that the tumor mutational burden is capable of sufficiently predicting long term clinical benefits (Champiat et al., 2014; Rizvi et al., 2015; Schumacher et al., 2015; Le et al., 2017). Moreover, recent studies have shown that higher somatic mutation and neoepitope loads have not been found in GBM cases responsive to ICIs (Zhao et al., 2019). The infiltration of mutation-reactive class I and class II T cells into the tumor seems not to be precluded by a low mutational load in GBM (Cloughesy et al., 2019; Schalper et al., 2019; Zhao et al., 2019). The presence of alterations in the *MMR* genes is another proposed biomarker (Cloughesy et al., 2019; Schalper et al., 2019). The expression of MHC class I molecules has been associated to responsiveness to ICIs since it is involved in the presentation of antigens and characterized by highly heterogeneous expression levels in GBM (Indraccolo et al., 2019).

## Discussion

Surgery followed by radiotherapy and chemotherapy with alkylating agents constitutes the standard first-line treatment of GBM (Stupp et al., 2005; Canoll and Goldman, 2008; Levine et al., 2015). Complete resection of the GBM tumors is generally not possible given its high invasive features. Although this combination therapy can prolong survival, the prognosis is still poor due to several factors including chemoresistance. Multiple mechanisms appear to be involved in the development of drug resistance in GBM including overexpression of drug efflux transporter pumps such as p-glycoprotein, the presence of a GSC population, a relevant activity of DNA repair mechanisms and dysregulated apotosis processes such as MGMT, the MMR pathway, the base excision repair (BER) pathway and the TP53 pathway (Walker et al., 1992; Bobola et al., 1996; Qian and Brent, 1997; Jaeckle et al., 1998; Chen et al., 1999; Esteller et al., 2000; Middlemas et al., 2000; Paz et al., 2004; Hegi et al., 2005; Helleday et al., 2005; Bryant and Helleday, 2006; Zawlik et al., 2009; van Nifterik et al., 2010; Malmstrom et al., 2012; Reifenberger et al., 2012; Armstrong et al., 2013; Brennan et al., 2013; Wiestler et al., 2013; Wick et al., 2014, 2018; Erasimus et al., 2016; Peng et al., 2016; Sun et al., 2018; Gupta et al., 2018; Zhang et al., 2018; Christmann and Kaina, 2019; Hafner et al., 2019; Mantovani et al., 2019). Tumor/TME interactions also contribute to the development of drug resistance in GBM tumor cells (Hanahan and Weinberg, 2011; Ab and Jn, 2012; Rodriguez-Hernandez et al., 2014; Munoz et al., 2015).

Systemic delivery uses existing vessels to deliver anti-tumor drugs to the tumor. To overcome the impediment of the BBB several strategies have been proposed including chemical modification of the drugs, high dose chemotherapy capable of inducing a transient BBB disruption, nanoparticle-based drug delivery and peptide-based drug delivery. Nevertheless, no straightforward results have still been reached (Siegal, 2013).

Glioblastoma stem cell cell population has been shown to induce a certain degree of radio- chemoresistance given their high expression of anti-apoptotic proteins, ATP-binding cassette pumps, their increased capability of DNA damage repair, as well as their high capacity of migration and invasion (Bao et al., 2006; Calabrese et al., 2007; Eyler and Rich, 2008; Diehn et al., 2009; Pietras et al., 2014). GSCs have been found capable of secreting angiogenic factors which in turn are responsible for an enhancement in the formation of tumor blood vessels, this has been frequently associated with high tumor aggressiveness. Moreover, the TME cell components can promote GSC survival by VEGF secretion (Wada et al., 2006). The interaction of TME with GSCs can facilitate tumor progression and consequently therapeutic resistance (Chen and Liu, 2012; Miura et al., 2013).

Over the past 10 years, the knowledge regarding genomic features of GBM has been greatly increased by comprehensive multiplatform genome-wide analyses. As a result of these analyses, it has emerged that GBM comprises a group of highly heterogeneous tumor types, each with peculiar molecular/genetic features (Tcga, 2008; Brennan et al., 2013; Wang et al., 2016).

In GBM the phase I/II and III trials investigating the use of therapies molecularly targeting oncogenic alterations did not generally show straightforward results and, consequently, their clinical utilization is still limited. However, although limited activity or no therapeutic efficacy has so far been produced by the use of TKIs, improvement in understanding the mechanisms of action of these compounds could help to determine how to better incorporate their use in the existing treatment modalities. Redundancies are frequently present in the molecular pathways that can be targeted which makes the inhibition of any pathway largely ineffective (Tcga, 2008; Brennan et al., 2013; Wang et al., 2016). The failure of targeted therapies can also be ascribed to another possible reason such as the fact that several genomic alterations are important only for the initial stages of tumor progression whereas other molecular mechanisms outweigh their role in the later stages. On the other hand, several genomic alterations in GBM can interfere with GBM cell metabolism. In particular, alterations in the growth factor signaling pathways that can control metabolic flux have been found in high frequency as well as recurrent mutations in *IDH1* and *IDH2* genes, whose encoded proteins are part of the tricarboxylic acid (TCA) cycle. Alterations of the cellular metabolism, which is controlled also by the biochemical microenvironment, could contribute to the failure of the proposed targeted therapies (Tcga, 2008; Brennan et al., 2013; Wang et al., 2016). A better understanding of the interactions constituting this interplay between altered genome and biochemical microenvironment could contribute to finding more effective treatment strategies in the reverting of altered cellular metabolism of GBM cells.

The TME of GBM is largely immunosuppressive, therefore efficiency of ICI treatments can be strongly affected by this condition (Akiyama et al., 2001; Brat and Van Meir, 2004; Brat et al., 2004; Nimsky et al., 2005; Zimmermann and Dours-Zimmermann, 2008; Persano et al., 2011; Sherriff et al., 2013; Dicker et al., 2014; Hambardzumyan and Bergers, 2015; Young et al., 2015; Chen and Hambardzumyan, 2018). GBM patients frequently present reduced levels of circulating CD4^+^ and CD8^+^ lymphocytes as a consequence of chemotherapy treatments (Gustafson et al., 2010; Mirzaei et al., 2017). A clear molecular/immunological signature that can be predictive of response to ICI treatments has not yet been identified (Motzer et al., 2015; Goldberg et al., 2016; Reck et al., 2016; Schwartz et al., 2016; Reiss et al., 2017; Reardon et al., 2018; Cloughesy et al., 2019; Schalper et al., 2019).

The treatment of different cancers has markedly been revolutionized by immunotherapy. Nevertheless, the data obtained so far concerning the use of ICIs for the treatment of GBM patients seem to be not sufficient to propose this type of immunotherapy as a standard treatment for GBM (Reardon et al., 2014, 2016; Motzer et al., 2015; Goldberg et al., 2016; Reck et al., 2016; Schwartz et al., 2016; Reardon et al., 2017; Reiss et al., 2017; Wen et al., 2018; Cloughesy et al., 2019; Schalper et al., 2019).

**FIGURE 3:**
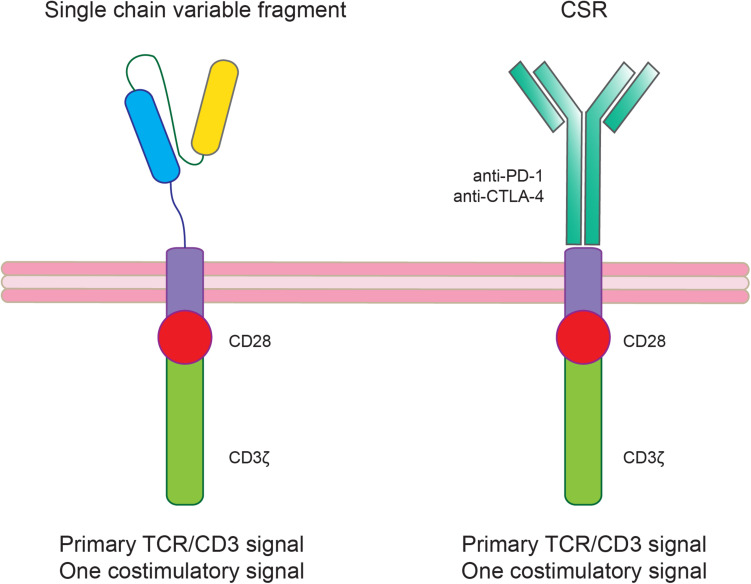
Modified chimeric antigen receptor-T (CAR-T) cells to ameliorate treatment efficacy by counteracting the immunosuppressive glioblastoma (GBM) tumor microenvironment (TME). The co-expression of an activating chimeric switch receptor (CSR), that combines the extracellular ligand-binding domain of an inhibitory receptor (PD-1 or CTLA-4) fused through a transmembrane domain with the cytoplasmic co-stimulatory signaling domain of CD28, could improve CAR-T cell efficacy in GBM.

Another immunotherapy approach that can be used also in combinations with ICIs is the chimeric antigen receptor-T (CAR-T) cell therapy targeting specific tumor associated antigens. The introduction of CAR-T cell therapy approaches also in solid tumors including GBM has been favored by the success of this therapy in hematological malignancies (Neelapu et al., 2017; Maude et al., 2018). Concerning GBM treatment, several clinical trials have been proposed showing that there are still substantial obstacles including TME immune suppression (Table 3; Morgan et al., 2010; Brown et al., 2015, 2016; Zah et al., 2016; Ahmed et al., 2017; Walseng et al., 2017; Richman et al., 2018). To increase CAR-T treatment efficacy several CAR-T modifications have been proposed such as the knocking out of genes encoding T cell inhibitory receptors or signaling molecules (e.g., PD-1 or CTLA-4) or the co-expression of activating chimeric switch receptor (CSR; Figure 3; Prosser et al., 2012; Shin et al., 2012; Ankri et al., 2013; Kobold et al., 2015; Liu et al., 2016).

**TABLE 3:** Clinical trials in glioblastoma (GBM) using chimeric antigen receptor-T (CAR-T).

Title	Condition or Disease	Intervention/Treatment	NCT Number	Status	Status Phase
Pilot study of autologous anti-EGFRvIII CAR-T cells in recurrent glioblastoma multiforme	Glioblastoma multiforme	Biological: anti-EGFRvIII CAR-T cells drug: cyclophosphamide Drug: Fludarabine	NCT02844062		Phase I
Pilot study of B7-H3 CAR-T in treating patients with recurrent and refractory glioblastoma	Recurrent glioblastoma Refractory glioblastoma	Drug: B7-H3 CAR-T Drug: Temozolomide	NCT04385173	Recruiting	Phase I
B7-H3 CAR-T for recurrent or refractory glioblastoma	Recurrent glioblastoma Refractory glioblastoma	Drug: Temozolomide Biological: B7-H3 CAR-T	NCT04077866	Not yet recruiting	Phase I, II
CD147-CAR-T cells in patients with recurrent malignant glioma	Recurrent glioblastoma CD147 positive	Biological: CD147-CAR-T	NCT04045847	Active, not recruiting	Early phase I
CART-EGFRvIII + pembrolizumab in GBM	Glioblastoma	Biological: CART-EGFRvIII T cells Biological: Pembrolizumab	NCT03726515	Active, not recruiting	Phase I
EGFRvIII CAR-T cells for newly diagnosed WHO grade IV malignant glioma	Glioblastoma Gliosarcoma	Biological: EGFRvIII CAR-T cells	NCT02664363	Terminated	Phase I
Chimeric antigen receptor (CAR- T) cells with a chlorotoxin tumor-targeting domain for the treatment of recurrent or progressive glioblastoma	Recurrent glioblastoma Recurrent malignant glioma recurrent WHO grade II glioma recurrent WHO grade III glioma	Biological: Chlorotoxin (EQ)-CD28-CD3zeta-CD19t-expressing CAR-TTlymphocytes NCI SYs	NCT04214392	Recluting	Phase I
IL13Ralpha2-targeted chimeric antigen receptor (CAR-T) T cells with or without nivolumab and ipilimumab in treating patients with recurrent or refractory glioblastoma	Recurrent glioblastoma Refractory glioblastoma	Biological: IL13Ralpha2-specific Hinge-optimized 4-1BB-co-stimulatory CAR/Truncated CD19-expressing autologous TN/MEM Cells Biological: Ipilimumab Biological: Nivolumab	NCT04003649	Recluting	Phase I
Autologous T cells redirected to EGFRVIII-with a chimeric antigen receptor in patients with EGFRVIII+ glioblastoma	Patients with residual or reccurent EGFRvIII+ glioma	Biological: CART-EGFRvIII T cells	NCT02209376	Terminated Result	Phase I
NKG2D-based CAR-T-cells Immunotherapy for patient with r/r NKG2DL+ solid tumors	Hepatocellular carcinoma Glioblastoma Medulloblastoma Colon cancer	Biological: NKG2D-based CAR-T-cells	NCT04270461	Not yet recruiting	Phase I
Pilot study of autologous chimeric switch receptor modified T Cells in recurrent glioblastoma multiforme	Glioblastoma multiforme	Biological: Anti-PD-L1 CSR T cells Drug: Cyclophosphamide Drug: Fludarabine	NCT02937844		Phase I
Intracerebral EGFR-vIII CAR-T cells for recurrent GBM	Recurrent glioblastoma Recurrent gliosarcoma	Biological: EGFRvIII-CARs	NCT03283631	Recluting	Phase I
Combination of immunization and radiotherapy for malignant gliomas (*In situ* Vac1)	High grade glioma Glioblastoma Glioma of brainstem Glioma Malignant	Combination Product: Combined immune adjuvants and radiation	NCT03392545	Recluting	Phase I
CAR-T cell receptor immunotherapy targeting EGFRvIII for patients with malignant gliomas expressing EGFRvIII	Malignant glioma Glioblastoma Brain cancer Gliosarcoma	Biological: Epidermal growth factor receptor (EGFRv)III Chimeric antigen receptor (CAR) transduced PBL Drug: Aldesleukin Drug: Fludarabine Drug: Cyclophosphamide	NCT01454596	Completed	Phase I, II
Immunogene-modified T (IgT) cells against glioblastoma multiforme	Glioblastoma multiforme of brain glioblastoma multiforme	Biological: Antigen-specific IgT cells	NCT03170141	Enrolling by invitation	Phase I
CMV-specific cytotoxic T lymphocytes expressing CAR-T targeting HER2 in patients with GBM (HERT-GBM)	Glioblastoma multiforme	Biological: HER.CAR-TCMV-specific CTLs	NCT01109095	Completed	Phase I
Genetically modified T-cells in treating patients with recurrent or refractory malignant glioma	Malignant glioma Refractory brain neoplasm Recurrent brain neoplasm Glioblastoma	Biological: IL13Rα2-specific, hinge-optimized, 41BB-costimulatory CAR/truncated CD19-expressing Autologous T lymphocytes Biological: Vaccine Therapy	NCT02208362	Recluting	Phase I
Memory-enriched T cells in treating patients with recurrent or refractory grade III-IV glioma	Glioblastoma HER2/Neu positive Malignant glioma Recurrent glioma Refractory glioma WHO grade III glioma	Biological: CD19CAR-CD28-CD3zeta-EGFRt-expressing Tcm-enriched T-lymphocytes Biological: CD19CAR-CD28-CD3zeta-EGFRt-expressing Tn/mem-enriched T-lymphocytes	NCT03389230	Recluting	Phase I

*Summarized in the Table are the ongoing clinical trials present on ClinicalTrials.gov searching the keywords “glioblastoma multiforme” and “CAR-T.” The research has been done adding the following filters: “Not yet recruiting”; “Recruiting”; “Enrolling by invitation”; “Active, not recruiting”; “Suspended”; “Terminate”; “Completed”; “Withdrawn”; “Unknown status”; “The research has been performed on October 21st, 2020.”*

Understanding the molecular and immunological complexity of GBM more and more could provide the grounds for the introduction of other immunotherapeutic approaches such as the use of CAR-T cell therapy, in combination with ICIs or TKIs, in the treatment paradigm of GBM.

## Author Contributions

All authors listed have made a substantial, direct and intellectual contribution to the work, and approved it for publication.

## Conflict of Interest

The authors declare that the research was conducted in the absence of any commercial or financial relationships that could be construed as a potential conflict of interest.
